# Benefits and Safety of Empiric Antibiotic Treatment Active Against KPC-Producing *Klebsiella pneumoniae* for Febrile Neutropenic Episodes in Colonized Children with Acute Leukemia—An 8-Year Retrospective Observational Study

**DOI:** 10.3390/antibiotics13111017

**Published:** 2024-10-29

**Authors:** Alessandra Micozzi, Cristina Luise, Chiara Lisi, Luisa Moleti, Stefania Santilli, Giuseppe Gentile

**Affiliations:** 1 Haematology, Department of Translational and Precision Medicine, Sapienza University of Rome, 00161 Rome, Italy; lisichiara92@gmail.com (C.L.); giuseppe.gentile@uniroma1.it (G.G.); 2 Haematology, Azienda Ospedaliera di Rilievo Nazionale Antonio Cardarelli, 80131 Naples, Italy; cristina.luise@aocardarelli.it; 3 Department of Haematology, Oncology and Dermatology, Azienda Policlinico Umberto I, 00161 Rome, Italy; moleti@bce.uniroma1.it; 4 Department of Diagnostics, Azienda Policlinico Umberto I, 00161 Rome, Italy; santilli@bce.uniroma1.it

**Keywords:** children, acute leukemia, febrile neutropenia, KPC-*Klebsiella pneumoniae*, empiric antibiotic treatment

## Abstract

In children with acute leukemia (AL), the mortality rate from *Klebsiella pneumoniae* carbapenemase (KPC)-producing *K. pneumoniae* bloodstream infection (KPC-KpBSI) exceeds 50%, highest when active treatment is delayed. Neutropenic KPC-*K. pneumoniae* carriers are at high risk of KPC-KpBSI, and preemptive empiric antibiotic treatment (EAT) of febrile neutropenic episodes (FNEs) active against KPC-*K. pneumoniae* may reduce this mortality. We conducted an 8-year (2014–2021) retrospective observational study of 112 febrile neutropenic episodes (FNEs) in 32 children with AL who were KPC-*K. pneumoniae* carriers: standard EAT for 39 FNEs and active EAT for 73 FNEs (52 ceftazidime/avibactam (CAZAVI)-based and 21 colistin-based combinations, and 5 CAZAVI monotherapy). Successful outcomes (survival from FNE) were observed in 94%; seven were fatal, with four due to infectious causes. KPC-KpBSIs caused 10/112 FNEs, 10/20 g-negative BSIs, and 3 deaths. The mortality rate of KPC-KpBSI was 30%. Active EAT was successful in 97% of the FNEs, compared to 87% with standard EAT. All deaths from KPC-KpBSI occurred in patients who received standard EAT, while none occurred with active EAT. KPC-KpBSI mortality rate with initial inactive treatment was 60%. CAZAVI-based EAT was successful in all FNEs, with a higher success rate without any modification compared to colistin-based EAT, where nephrotoxicity occurred in 14%. Therefore, active EAT, mainly a CAZAVI-based combination, was effective, safe, and associated with low overall and KPC-KpBSI-related mortality.

## 1. Introduction

Infections caused by carbapenemase-producing Enterobacteriaceae (CPE) pose a significant public health concern [1,2,3,4,5,6,7,8,9,10,11,12,13,14,15,16,17,18]. While most studies have focused on CPE infections predominantly in adults [2,18], data on the prevalence and pathogenesis of *Klebsiella pneumoniae* carbapenemase (KPC)-producing *K. pneumoniae* (a type of CPE) in hospitalized children with hematological malignancies remain scarce [6,13,15,18,19]. Approximately a decade ago, some differences in CPE infections between adult and pediatric populations were observed. In Europe, between 2011 and 2012, the prevalence of carbapenem resistance (CR) among *Klebsiella pneumonia* blood isolates was higher in adults (13.5%) than in children (6.5%) [20]. In cancer patients, between 2005 and 2011, a lower prevalence of CR-resistant blood isolates was found in the pediatric population compared to adults [21]. A 2013 point-prevalence study of CPE colonization in high-risk children conducted in Greece found a low rate of colonization, despite CPE being endemic in adults [4].

Similar to the adult population, KPC-*K. pneumoniae* colonization and infections in children and its nosocomial spread in pediatric hematology-oncology centers increased, especially in countries with high endemicity [1,5,7,9,10,12,13,14,17,19]. Hematologic and oncologic diseases are recognized as risk factors for CPE infections [1,3,6,7,11,12,19].

Mortality rates among children with CPE infections range from 8% to 52% [1,3], lower than those reported in adults. These rates vary based on age, the source of infection (with higher rates in bloodstream infections (BSI)) [3,6,11,12,14,15], and the presence of hematologic or oncologic diseases, which are associated with high fatality rates [1,3,12,14,16]. For adults with hematological malignancies [22,23,24], initial effective antibiotic treatment is essential for survival from KPC-*K. pneumoniae* bloodstream infections (KPC-KpBSI). High rates of KPC-KpBSI-related mortality have been reported in children with hematological malignancies who received delayed antibiotic treatment [6,7,10,11,12,14,15,25].

Colonization by KPC-*K. pneumoniae* is a recognized predictive factor for KPC-KpBSI [1,2,4,6,7,8,9,10,11,12,16,19,22,23,24], particularly in patients with acute leukemia undergoing intensive chemotherapy and/or hematopoietic stem cell transplant (HSCT). The 2013 European Conference on Infections in Leukemia (ECIL) guidelines [26] for adult patients with febrile neutropenia and the 2020 ECIL guidelines [27] for pediatric patients with cancer or those post-HSCT suggest that empiric antibiotic treatment (EAT) should be adjusted for patients currently colonized or previously infected with resistant Gram-negative bacteria or in healthcare centers with a high rate of resistant pathogens. However, due to the limited data available on the safety and efficacy of new drug combinations (e.g., novel β-lactam/β-lactamase combinations) in pediatric patients, the 2020 ECIL guidelines [27] do not recommend their routine use as EAT.

Based on the significant reduction in overall and KPC-KpBSI-related mortality seen in adults with hematological malignancies who are KPC-producing *K. pneumoniae* carriers [23], we extended the preemptive use of active EAT against KPC-*K. pneumoniae* in children with acute leukemia presenting with febrile neutropenia episode (FNE) and colonized by KPC-producing *K. pneumoniae* since 2014. In this retrospective observational study, we investigated the benefits and safety of antibiotics active against KPC-*K. pneumoniae*, ceftazidime/avibactam (CAZAVI), or colistin-based treatments compared to first-line standard antibiotics, piperacillin-tazobactam monotherapy, or combined with tigecyclin, for the empiric treatment of FNEs in children with acute leukemia who were identified as KPC-producing *K. pneumoniae* carriers. Additionally, we compared the empiric use of CAZAVI and colistin.

## 2. Results

### 2.1. Characteristics of FNEs Treated with Standard and Active EAT

The majority of FNEs (65%; 73/112) occurred in children with acute lymphoblastic leukemia, while the remaining (35%; 39/112) occurred in those with acute myeloid leukemia. Many (53%) of the FNEs occurred in patients during hospitalization for intensive chemotherapy, 14% of FNEs in allogenic HSCT recipients, and 33% in those managed as outpatients who were subsequently hospitalized for the FNE (Table 1). A total of 22 of 32 (64%) children were empirically treated for more than one FNE occurred during different chemotherapy courses (mean 4.5 FNE, range 2–14).

The site of KPC-*K. pneumoniae* colonization was observed in the rectum in 96% of cases. Seventy-nine percent (88/112) of FNEs developed in patients who were KPC-*K. pneumoniae* carriers at the time of hospital admission (6 median days; range 1–26 days). Twenty-one percent of children developed new colonization during their hospital stay.

Fever occurred during profound neutropenia in 64 (57%) patients, 12 (11%) presented with shock, and 7 patients were admitted to the pediatric intensive care unit (PICU).

A BSI was documented in 42 (37.5%) FNEs, with 20 BSIs (48%) due to Gram-negative bacteria, and KPC-*K. pneumoniae* was identified in 10 cases (24% of all BSI, 50% of Gram-negative bacteria BSI, and 9% of all FNEs). KPC-KpBSI caused 15% (6/39) of FNEs to develop in children with acute myeloid leukemia and 5% (4/73) in those with acute lymphoblastic leukemia (*p* = 0.009).

Overall, a successful response was observed in 94% of the 112 FNE cases, with fever resolving within 72 h in 80 (71%) cases.

Seven FNEs (6.2%) had a fatal outcome, with four due to infectious causes (3.5% of FNEs, 12.5% of patients). Two cases resulted in death within 4 days (1.7% of FNEs). Three children died from KPC-KpBSI; all were transferred to the PICU; two with septic shock died after 4 and 13 days; and one child with acute lymphoblastic leukemia relapse developed pneumonia and respiratory failure and died on day 30. Overall, the mortality rate of KPC-KpBSI was 30% (3/10 KPC-KpBSI were fatal) (Table 2). Death due to KPC-KpBSI occurred in 9.3% (3/32) of children with acute leukemia who were KPC-*K. pneumoniae* carriers and in 2.6% (3/112) of FNE cases. One patient died of *Pseudomonas aeruginosa* BSI on day 4. Three children died due to unmanaged leukemia.

### 2.2. Comparison Between Standard and Active EAT

Thirty-nine (35%) and seventy-three (65%) FNEs were handled with standard EAT and EAT active against KPC-*K. pneumoniae* (active EAT), respectively. For standard EAT, piperacillin-tazobactam was used as monotherapy in 18 cases (46%) and combined with tigecycline in 21 (54%). Active EAT consisted of antibiotic combinations in 68/73 (93%) cases (CAZAVI-based combination in 52 (46%), colistin-based combination in 21 (19%), the combinations included tigecycline and gentamicin in 44 (60%)). CAZAVI monotherapy was used in five FNE cases.

Overall, successful outcomes with survival from an FNE were achieved in 87% (34/39) and 97% (71/73) of patients treated with standard and active EAT, respectively (*p* = 0.004). Three of the five deaths observed in the standard EAT group were due to KPC-KpBSI (3/39 FNEs; 7.7%). One *P. aeruginosa* BSI-related death was observed in the active EAT group (1/73 FNEs; 1.3%) (Table 2).

Comparing FNEs treated with standard EAT and active EAT, no significant differences were seen based on sex, age, severity of neutropenia, or duration of profound neutropenia (Table 1). A total of 14 of the 22 children (64%) empirically treated for more than one FNE developed during different chemotherapy courses received both standard EAT and active EAT in different periods, without difference in the overall efficacy of the EAT. In particular, two of the four patients that died of infective causes had received both standard EAT and active EAT.

We observed a trend towards more frequent use of active EAT in children with acute myeloid leukemia (active EAT in 30/39 (77%) and 43/73 (59%) in children with acute myeloid leukemia and acute lymphoblastic leukemia, respectively; *p* = 0.06). Active EAT was used in 75% (9/12) of patients who presented with shock compared to 25% (3/12) of patients who were treated with standard EAT (*p* = 0.03), modified within 24 h with antibiotics effective against KPC-*K. pneumoniae* in all the cases. Forty-one percent of standard EAT was administered to children managed as outpatients and hospitalized for an FNE, compared to 29% administered with active EAT for this purpose (*p* = 0.03).

As shown in Table 2, no differences emerged in the success rate with or without EAT modification between the standard and active EAT groups nor in the reasons for EAT modification. In the standard EAT group, initial antibiotics were replaced with antibiotics effective against KPC-*K. pneumoniae* in 8 (20%) cases, while in the active EAT group, treatment targeting KPC-*K. pneumoniae* was implemented in 13 patients (18%) (Table 2). Adverse events were observed in five (7%) patients who received active EAT, whereas none were observed with standard EAT (Table 2).

Five patients with KPC-KpBSI initially received standard EAT (modified with targeted antibiotics within 24 h in all cases), and three of these cases were fatal (KPC-KpBSI mortality of 60% with initial non-targeted treatment). In contrast, five patients with KPC-KpBSI received active EAT, and all patients survived (*p* = 0.04).

Comparing CAZAVI and colistin-based EATs (Table 3), all FNEs treated with CAZAVI-based EAT had successful outcomes, while one death from *P. aeruginosa* BSI occurred in the colistin-based EAT group. Notably, CAZAVI-based EAT achieved a higher success rate without modification than colistin-based EAT (40/52 (77%) vs. 11/21 (52%), respectively; *p* = 0.05), and the combination of tigecycline and gentamycin with CAZAVI was successful without modification in 89% of cases (combined with colistin in 56%; *p* = 0.02) (Table 3). Nephrotoxicity requiring treatment discontinuation occurred in 3/21 (14%) patients receiving colistin-based EAT (none in CAZAVI-based EAT, *p* = 0.02), and two cases of cutaneous rashes (4%) occurred in children treated with CAZAVI; these resolved after discontinuation in all cases.

## 3. Discussion

CPE infections are difficult to treat, leading to high mortality rates, especially in vulnerable populations such as neutropenic children with acute leukemia. This study confirms that in patients with febrile neutropenia, the susceptibility of Gram-negative bacteria to initial EAT is crucial for successful treatment during hematological malignancies [28]. Our retrospective observational study addressed the overall benefits of EAT effective against KPC-*K. pneumoniae* for managing FNEs in children with acute leukemia who were KPC-*K. pneumoniae* carriers. Overall, KPC-KpBSI was documented in 9% of FNEs in colonized children, with death due to KPC-KpBSI occurring in 7.6% of FNEs. KPC-KpBSI caused 3 of 4 deaths from primary infection and resulted in the deaths of 3 of 32 (9.3%) colonized children. Active EAT was successful in 97% of the FNEs and all KPC-KpBSI cases. FNEs empirically treated with standard antibiotics were fatal in 13% of cases; KPC-KpBSI was fatal in 7.7%, with a 60% mortality rate for KPC-KpBSI treated initially with non-targeted EAT.

There has been a steady increase in KPC-*K. pneumoniae* colonization and infection in children, particularly in countries with high endemicity [1,2,4,6,7,8,9,10,11,12,16,19]. From 2013 to 2017, in an Italian pediatric hospital, CPE represented 3.5% of all invasive infections caused by Enterobacteriaceae, with overall rates increasing from 83.03 to 191.34 for hospital discharges and from 1.03 to 2.06 for hospitalization days [17]. A two-year (2012–2013) nationwide survey in Italy of pediatric hematology-oncology centers observed a threefold increase in colonization rates and a fourfold increase in BSI caused by CPE, with a 90-day mortality rate of 14% [10].

Available data suggest that CPE risk factors and outcomes in the pediatric population mirror trends observed in studies on adult populations. CPE infections occur predominantly in critically ill pediatric patients with serious underlying conditions [1,3,6,7,10,11,14,19], such as children with hematological malignancies. A prospective 18-month survey conducted in Italy between 2012 and 2013 on 15 children with CPE isolated from clinical specimens (4 BSI; 27%) reported that 73% were from the hematology-oncology unit and 10 patients had hematological malignancies [7]. In pediatric patients with cancer, high-intensity chemotherapy and isolation of antibiotic-resistant Gram-negative bacteria from any site within the preceding 12 months were independently associated with antibiotic-resistant Gram-negative bacteria BSI [6]. Among 50 CPE-BSIs reported in India [14], 46% of children had hematological disease, 32% had malignancy, 44% were neutropenic, and 80% of the CPE-BSIs developed on antibiotic treatment, with meropenem used in half of the cases.

In this study, KPC-KpBSI represented 50% of all documented Gram-negative bacteria-BSIs and caused 9% of FNEs. We previously reported that KPC-KpBSI caused nearly a quarter of FNEs that developed in adults with AL colonized by KPC-*K. pneumoniae* [24]. The different characteristics of the two populations might explain the lower rate (9%) found in this study, with 65% of FNEs occurring in children with acute lymphoblastic leukemia, whereas, in our previous study, 71% of FNEs occurred in adults with acute myeloid leukemia with longer chemotherapy-related neutropenia and more severe gastrointestinal toxicity. However, despite the small sample size, we observed that KPC-KpBSI caused FNEs in 15% of children with acute myeloid leukemia and 5% of children with ALL.

CPE infections in the pediatric population increase the risk of mortality 6- to 11-fold compared to non-CPE infections. Mortality rates of CPE infections range from 8% to 52% [14], with bacteremia associated with the highest crude mortality rate, especially in children with cancer or who are immunosuppressed [3,12,14]. A retrospective multicenter Italian study [12] reported a 23.5% mortality rate in 34 children with CPE infections (41% BSI), immunosuppressed in half of the cases, with all deaths due to CPE-related causes, with oncologic or immunosuppressive conditions associated with a fatal outcome. In 34 children with carbapenem-resistant (CR)-*K. pneumoniae* infection (79% immunosuppressed), Díaz et al. [11] reported an overall mortality rate of 38%, with a CR-*K. pneumoniae* BSI mortality rate of 37.5%. In our study, 9.3% (3/32) of the children with acute leukemia who were colonized by KPC-*K. pneumoniae* died from KPC-KpBSI, and the KPC-KpBSI mortality was 30% in children with acute leukemia who received active EAT in the 50% of cases.

Initial active treatment is essential for KPC-KpBSI survival [22,23,24,25]. In colonized patients with acute leukemia at high risk of KPC-KpBSI, empirical treatment of FNEs with targeted antibiotics can ensure prompt therapy for KPC-KpBSI and protect patients from KPC-KpBSI-related deaths [23,29]. In our study, active EAT, typically a combination of antibiotics, was successful in all KPC-KpBSI cases, confirming the better outcomes for KPC-KpBSI in children who received prompt treatment targeting gut colonization [6,7,12,14].

Inappropriate initial treatment has been associated with higher mortality among pediatric patients with CPE-BSIs [6,11,14,15,18,25]. Díaz et al. [11] reported that an effective agent was included in the initial therapy in only 47.3% of cases, with guided antimicrobial therapy starting on average 4 days later, resulting in 100% mortality without effective treatment. An Indian study [14] reported 85% mortality with failure to clear CR-BSI and significantly lower mortality rates when two or more effective drugs were used in combination.

In our study, KPC-KpBSI initially treated with non-targeted antibiotics was fatal in 60% of the cases, despite early modification to targeted treatment, similar to the mortality rates reported in adult patients with HM not receiving targeted treatment at the onset of BSI [22,23].

During the 8-year study, the active EATs changed from colistin to CAZAVI, making it difficult to accurately compare the standard and active EAT groups. This, together with the monocentric design, the low number of patients and FNE included, and the availability of CAZAVI only in 2018 represent a limitation to the present report, and prospective larger studies are needed to confirm and generalize the results.

Treatment of KPC-*K. pneumoniae* is, however, more complicated in children than in adults [18,29]. Specific recommendations are based on studies that include only adults [30], and combination therapies associated with better outcomes in adults [22,23,30] have not been extensively evaluated in children [14,18,29]. Data regarding the safety and optimal dosage of old and new antibiotics in children, as well as indications for the use of many new drugs, are scarce due to limited experience in pediatric patients.

Since 2013, we have preemptively used EAT against KPC-*K. pneumoniae* in adult patients with acute leukemia and febrile neutropenia colonized with KPC-*K. pneumoniae*. This approach was found to be safe and effective and was associated with low overall and KPC-KpBSI-related mortality [23]. Since 2014, we have gradually extended this strategy to children with febrile neutropenia and acute leukemia colonized with KPC-*K. pneumoniae.*

Colistin (alone or in combination) was the only available antibiotic against KPC-*K. pneumoniae*, and the lack of specific pediatric guidelines published since 2020 led us to be cautious until CAZAVI was approved for use in children. Colistin nephrotoxicity is reported in 8–19% of patients, more frequently in critically ill children receiving a concomitant nephrotoxic agent [18,31], which is frequent in children treated for acute leukemia. Colistin nephrotoxicity occurred in 14% of cases in our study and was higher than the 8% reported in adults with acute leukemia [24].

In our experience, clinicians are more inclined to use active EAT (even when colistin was the only available choice) in children with febrile neutropenia with KPC-*K. pneumoniae* carriers evaluated as high risk: children with acute myeloid leukemia undergoing intensive chemotherapy, allogeneic HSCT recipients, and/or those facing severe clinical conditions, such as septic shock. The standard EAT was used more frequently in colonized children evaluated as having a lower risk and better clinical conditions, such as those managed as outpatients until hospitalization for an FNE. CAZAVI is increasingly used for the treatment of KPC-*K. pneumoniae* infections in adult patients with HMs, representing an effective therapeutic alternative with increased efficacy and low toxicity [23,24,26,32]. Currently, clinical data on the treatment of CPE infections with CAZAVI in the pediatric population are limited. In agreement with other clinical data [15,18,25,33,34], in our study, CAZAVI was safe and well tolerated. Although the small sample size did not allow for significant conclusions, CAZAVI-based EAT was successful in all FNEs and Gram-negative bacteria-BSI cases, with CAZAVI combined with tigecycline and gentamycin successful without modification in 86% of FNEs. Recently, concerns have been raised regarding the emergence of CAZAVI resistance [34] reported during [35] and independently [36] from previous ceftazidime-avibactam exposure.

Its use is strictly reserved for select high-risk patients, and combination therapy might be an option to avoid the emergence of resistance.

## 4. Materials and Methods

### 4.1. Study Characteristics and Ethics Approval

This retrospective observational study, conducted between June 2014 and September 2021 at the Hematology Department of Sapienza University of Rome (Italy), analyzed 112 FNEs in 32 pediatric patients with acute leukemia colonized by KPC-*K. pneumoniae.* Standard EAT and EAT active against KPC-*K. pneumoniae* (active EAT) were compared. The study was approved by the Institutional Review Board and the internal ethics committee of the Department of Translational and Precision Medicine. The ethics committee waived the need for informed consent. Patient data were obtained from the medical records of the patients stored at the institutional repository, and each patient included in the study was assigned a code for subsequent analysis. The data were analyzed using an anonymized database, in compliance with the Declaration of Helsinki.

### 4.2. Data Collection

Data on age, sex, acute leukemia characteristics, receipt of chemotherapy, neutropenia (<1000 neutrophils/mmc) and profound neutropenia (<100 neutrophils/mmc) days, clinical presentation of the FNE and EAT, microbiological documentation, clinical response to EAT, outcomes, adverse events, and toxicity to EAT were recorded. KPC-*K. pneumoniae* colonization in children was identified from rectal swabs collected before hospitalization, upon admission, and weekly during the course of the hospital stay. Response was evaluated on day 4 after EAT completion (early evaluation), day 14 after completion, and after recovery from neutropenia (overall evaluation).

### 4.3. Study Outcomes

We evaluated a successful outcome as survival from an FNE. Failure was recorded when a patient died of any cause. We also compared standard and active EAT to evaluate differences in (A) mortality from primary infection and early death (fatality within 2 weeks from the commencement of treatment); (B) successful outcomes both overall and without EAT modification; (C) clinical deterioration (shock, acute respiratory distress syndrome, or multiple organ failure); and (D) toxicity requiring treatment interruption (renal failure was defined as a serum creatinine level ≥ 2 mg/dL with or without renal replacement therapy).

### 4.4. Standard and Active EAT

Standard EAT included piperacillin-tazobactam (<12 years old: 100 mg/kg and 12.5 mg/kg, every 8 h; >12 years old: 4 g/kg and 0.5 g/kg, every 8 h) with or without tigecycline (<12 years old: 1.2 mg/kg, every 12 h; >12 years old: 100 mg/kg loading dose, then 50 mg/kg, every 12 h). Active EAT included colistin (<40 kg: 75–150 MU/kg every 8 h, >40 kg: 4.5 MU every 8 h) combined with tigecycline and/or gentamicin (7 mg/kg/day, single dose). From December 2018, CAZAVI (6 months–18 years: 50 mg/kg and 12.5 mg/kg, every 8 h) monotherapy, or in combination with tigecycline with or without gentamycin, was administered to the patients. In Italy, CAZAVI was available for routine clinical use in adults from March 2018. In our Hematology Department, the children received CAZAVI therapy within the bounds of compassionate use programs set forth by the drug manufacturer (Pfizer) after approval from the ethics committee and written informed consent from each patient’s tutor from December 2018. The drug manufacturer did not influence the study or the analysis of the results.

### 4.5. Statistical Analysis

The χ-square test with a correction for continuity and Fisher’s exact test were used, when appropriate. The Wilcoxon test was used to compare mean values (Dotmatics-GraphPad).

## 5. Conclusions

KPC-*K. pneumoniae* poses a serious concern in children with neutropenia and acute leukemia. Colonization by KPC-*K. pneumoniae* is a major risk factor for KPC-KpBSI. Identifying colonization can be an effective tool for managing children with hematological malignancies presenting with febrile neutropenia who require EAT targeting potential multidrug-resistant pathogens. This approach can reduce the rates of non-targeted EAT and BSI-related mortality. In our experience, EAT targeting KPC-*K. pneumoniae* was effective, safe, and associated with low overall and KPC-KpBSI-related mortality. Our findings support the routine use of active EAT in febrile neutropenic children with acute leukemia who are KPC-*K. pneumoniae* carriers.

## Figures and Tables

**Table 1:** Characteristics of 112 febrile neutropenic episodes treated with empiric antibiotic treatment in children with acute leukemia who are *Klebsiella pneumoniae* carbapenemase (KPC)-producing *K. pneumoniae* carriers.

	FNEs n = 112	Standard EAT n = 39	Active EAT n = 73	*p*-Value
**Male/Female**	64/48	20/19 (49%)	28/45 (62%)	0.2
**Mean age, years (range)**	14 (2–18)	12 (2–17)	15 (3–18)	0.8
**Acute myeloid leukemia**	39 (35%)	9 (23%)	30 (41%)	0.06
**Acute lymphoblastic leukemia**	73 (65%)	30 (77%)	43 (59%)	
**Reason for hospitalization**				
Chemotherapy	59 (53)	20 (51%)	39 (53%)	0.6
Allogeneic HSCT	16 (14)	3 (8%)	13 (18%)	0.1
Febrile neutropenia episodes	37 (33)	16 (41%)	21 (29%)	0.03
**Total duration of neutropenia episode**				
median days with <1000 neutrophils/mm^3^ (range)	7.5 (3–90)	6 (3–82)	9 (6–90)	0.4
median days with <100 neutrophils/mm^3^ (range)	4.5 (0–69)	5 (0–67)	4 (0–68)	0.1
**<100 neutrophils/mm^3^ at febrile neutropenia onset**	64 (57%)	20 (51%)	44 (60%)	0.4
**Shock at febrile neutropenia onset**	12 (11%)	3 (8%)	9 (12%)	0.5
**Empiric antibiotic treatment**				
**Monotherapy**	23 (21%)	18 (46%)	5 (7%)	0.0001
Piperacillin/tazobactam		18 (46%)		
Ceftazidime/avibactam			5 (7%)	
**Combination regimens**	89 (79%)	21 (54%)	68 (93%)	0.0001
Including tigecycline	45 (40%)	21 (54%)	24 (33%)	0.042
Piperacillin/tazobactam plus tigecycline		21 (54%)		
Ceftazidime/avibactam plus tigecycline			17 (23%)	
Colistin plus tigecyclin			5 (7%)	
Including tigecycline and gentamicin	44 (39%)	0	44 (60%)	0.0001
Colistin plus tigecyclin plus gentamicin			16 (22%)	
Ceftazidime/avibactam plus tigecyclin plus gentamicin			28 (38%)	

FNE—febrile neutropenic episode, EAT—empiric antibiotic treatment, HSCT—hematopoietic stem cell transplant.

**Table 2:** Response to standard and active EAT.

	Total EAT n = 112	Standard EAT n = 39	Active EAT n = 73	*p*-Value
**Overall successful outcome** (survival from FNE)	105 (94%)	34 (87%)	71 (97%)	0.004
**Failure (death)**	7 (6.2%)	5 (12.8%)	2 (2.7%)	
Death from primary infection	4 (3.5%)	3 (7.6%)	1 (1.3%)	0.1
*KPC K. pneumoniae* BSI	3 (2.6%)	3 (7.6%)	0	0.04
Other Gram-negative bacteria BSI	1	-	1	
Death within 4 days	2 (1.7%)	1	1	
Death for underlying leukemia	3 (2.6%)	2 (5.1%)	1(1.3%)	
**Bloodstream infections:success of total**	42 (37.5%)	17 (44%)	25 (34)	
Other Gram-negative bacteria BSI	16 of 20 (80%)	5 of 8 (62%)	11 of 12(92%)	0.1
*KPC*-*K. pneumoniae* BSI	7 of 10 (70%)	2 of 5 (40%)	5 of 5 (100%)	0.1
Gram-positive BSI	22 of 22 (100%)	9 of 9 (100%)	13 of 13 (100%)	
**Success without EAT modification**	77 (69%)	26 (67%)	51 (70%)	0.8
Piperacillin/tazobactam monotherapy		10 of 18 (56%)		
Ceftazidime/avibactam monotherapy			3 of 5 (60%)	
Piperacillin/tazobactam plus tigecyclin		16 of 21 (76%)		
Colistin plus tigecyclin			2 of 5 (40%)	
Ceftazidime/avibactam plus tigecyclin			12 of 17 (71%)	
Combination including tigecycline and gentamicin			34 of 44 (77%)	
**Success with EAT modification**	28 (25%)	8 (21%)	20 (27%)	0.49
- Antibiotics effective against KPC-*K. pneumoniae* initiation		8 (21%)		
- Implementation against KPC-*K. pneumoniae*			13 (18%)	
**Reasons for modification**				
Fever persistence	23 of 28 (82%)	8 of 8 (100%)	15 of 20 (75%)	1
Clinical deterioration, shock	10 of 28 (35.7%)	3 of 8 (37.5%)	7 of 20 (35%)	1
Adverse events	5 of 28 (18%)	0	5 of 20 (25%)	0.05
**Modification within 24 h**	13 (12%)	8 (21%)	5 (7%)	0.058
Overall adverse events	5 (4%)	0	5 (7%)	0.1
Afebrile within 72 h	80 (71%)	23 (59%)	57 (78%)	0.3

EAT—empiric antibiotic treatment, FNE—febrile neutropenic episode, BSI—bloodstream infection, KPC-*K. pneumoniae*—*Klebsiella pneumoniae* carbapenemase (KPC)-producing *K. pneumoniae*.

**Table 3:** Comparison between ceftazidime/avibactam (CAVAZI)-based and colistin-based active empiric antibiotic treatment (EAT).

	CAZAVI-Based EAT n = 52	Colistin-Based EAT n = 21	*p*-Value
**Overall successful outcome** (survival from FNE)	52 (100%)	19 (90.4%)	0.07
**Death for primary infection**	0	1 (4.7%)	0.2
KPC-*K. pneumoniae* BSI	0	0	
Other Gram-negative bacteria BSI	0	1	
Death within 7 days	0	1	
**Gram-negative bacteria BSI** (success of total)	6 of 6	5 of 6	
KPC-*K. pneumoniae* BSI	2 of 2	3 of 3	
**Success without EAT modification**	40 (77%)	11 (52%)	0.05
combination including tigecycline and gentamycin	25 of 28 (89%)	9 of 16 (56%)	0.02
**Success with EAT modification**	12 (23%)	8 (38%)	0.2
Reasons for modification			
Fever persistence	5 of 12 (42%)	1 of 8 (12.5%)	0.3
Clinical deterioration, shock	5 of 12 (42%)	4 of 8 (50%)	1
Adverse events	2 of 12 (17%)	3 of 8 (37.5%)	0.3
**Adverse events**	2 (4%)	3 (14%)	0.1
Nephrotoxicity	0	3	0.6
Cutaneous rash	2	0	1

EAT—empiric antibiotic treatment, FNE—febrile neutropenic episode, BSI—bloodstream infection, KPC-*K. pneumoniae*—*Klebsiella pneumoniae* carbapenemase (KPC)-producing *K. pneumoniae*.

## Data Availability

Data is contained within the article.

## Abbreviations

CPE: carbapenemase-producing Enterobacteriaceae; CR: carbapenem resistance; KPC-producing *K. pneumoniae: Klebsiella pneumoniae* carbapenemase (KPC) producing *K. pneumoniae;* BSI: bloodstream infection; KPC-KpBSI: KPC-*K. pneumoniae* bloodstream infection; FNE febrile neutropenia episode; HSCT: hematopoietic stem cell transplant; EAT: empiric antibiotic treatment; CAZAVI: ceftazidime/avibactam.
